# Sterile Intraocular Inflammation Associated With Faricimab

**DOI:** 10.1001/jamaophthalmol.2024.3828

**Published:** 2024-10-10

**Authors:** Mariano Cozzi, Alexander Ziegler, Katrin Fasler, Daniel R. Muth, Frank Blaser, Sandrine A. Zweifel

**Affiliations:** 1 Department of Ophthalmology, University Hospital Zurich, University of Zurich, Zurich, Switzerland; 2 Eye Clinic, Department of Biomedical and Clinical Sciences, University of Milan, Milan, Italy; 3 Department of Clinical Neuroscience, Karolinska Institutet, Stockholm, Sweden; 4 St Erik Eye Hospital, Solna, Sweden

## Abstract

**Importance:**

Randomized clinical trials are conducted to establish both drug safety and efficacy. However, evidence of adverse events associated with these drugs in the clinical practice setting can be of value at generating hypotheses regarding less common safety issues, even if causality cannot be determined.

**Objective:**

To present and analyze cases of intraocular inflammation associated with faricimab therapy in patients referred to a single European institution.

**Design, Setting, and Participants:**

This was a review starting in April of 2024 of an observational case series. Patients were from a single academic-based tertiary referral center in Switzerland. Included in the analysis were patients referred for intraocular inflammation soon after receiving a faricimab intravitreal injection between June 1, 2022, and March 5, 2024.

**Exposure:**

Faricimab, 6 mg (0.05 mL of a 120-mg/mL solution), administrated for neovascular age-related macular degeneration or diabetic macular edema.

**Main Outcomes and Measures:**

The systemic and ocular histories and imaging data available were reviewed. The following were evaluated: visual acuity measured with habitual correction using the Early Treatment of Diabetic Retinopathy Study charts before and after the event; intraocular pressure; patient symptoms; anterior, intermediate, or posterior location of the intraocular inflammation; and the presence of retinal vasculitis. Multimodal imaging including color fundus photographs, fluorescein angiograms, indocyanine green angiograms, and optical coherence tomography were reviewed.

**Results:**

A total of 12 eyes from 7 patients (mean [SD] age, 73.3 [16.7] years; 4 female [57.1%]) over 22 months were identified as having noninfectious intraocular inflammation after intravitreal faricimab injections. Among these cases, in 2 eyes, retinal vasculitis was present together with anterior and posterior inflammation. One of the 2 eyes had an occlusive form of vasculitis of the arteries and veins, leading to subsequent macular capillary nonperfusion and clinically relevant irreversible vision deterioration from 20/80 to 20/2000. The remaining eyes were characterized by moderate anterior segment inflammation without substantial vision changes. The intraocular inflammation event occurred after a median (IQR) of 3.5 (2.0-4.3) faricimab injections. The median (IQR) interval between the last faricimab injection and the diagnosis of inflammation was 28 (24-38) days. Increased intraocular pressure of 30 mm Hg or higher was found in 3 eyes.

**Conclusions and Relevance:**

This case series highlights the occurrence of rare, but potentially severe, intraocular inflammation associated with faricimab therapy. Although these findings do not prove causality and can only generate hypotheses for future investigations, these results suggest the importance of continuous surveillance and monitoring for patients undergoing faricimab therapy to promptly identify and manage potential adverse events.

## Key Points

**Question:**

What are some characteristics and outcomes of sterile intraocular inflammation after injection of faricimab?

**Findings:**

In a case series study from 1 institution over 22 months, 12 eyes of 7 patients presented with intraocular inflammation associated with faricimab intravitreal injections. Among these, 2 eyes developed retinal vasculitis; 1 of 2 eyes with vasculitis showed an occlusive form of vasculitis involving both arteries and veins, with subsequent macular capillary nonperfusion and clinically relevant irreversible vision loss.

**Meaning:**

These results suggest the importance of maintaining ongoing vigilance for potential sight-threatening intraocular inflammation after intravitreal faricimab administration.

## Teaser

This case series study evaluates the characteristics and outcomes of sterile intraocular inflammation after injection of faricimab to treat neovascular age-related macular degeneration or diabetic macular edema.

## Introduction

Faricimab, a humanized, bispecific, immunoglobulin G (IgG) monoclonal antibody, has received approval as an intravitreal injection (IVI) from the US Food and Drug Administration for addressing neovascular age-related macular degeneration (AMD), diabetic macular edema (DME), and macular edema secondary to retinal vein occlusion.^1,2,3,4,5^ This therapeutic molecule is designed to target the vascular endothelial growth factor A (VEGF-A) and the angiopoietin 2 pathways, aiming to enhance vascular stability while mitigating neovascularization and hyperpermeability. Multiple multicenter randomized clinical trials (RCTs) have demonstrated positive visual outcomes, suggesting potential extended intervals between treatments for patients receiving faricimab. The safety profile has been also investigated and compared with aflibercept, 2 mg. Overall, in the registration process, faricimab exhibited an acceptable safety profile and was generally well tolerated.^2,3^

Various VEGF inhibitors have received approval and are now considered the standard of care for neovascular AMD treatment. However, RCTs are primarily designed to define the drug’s efficacy and are not sufficiently powered to detect rare adverse events. Phase 4 studies, supported by clinical practice setting evidence (ie, real-world studies), serve as enduring tools for monitoring the incidence and prevalence of both ocular and systemic adverse events.^6,7,8,9^ Nevertheless, in the immediate aftermath of approval by national regulatory agencies, clinical case reports offer a timely and current overview of the drug’s safety profile.

Intraocular inflammation (IOI) is a potential adverse event associated with IVI of anti-VEGF agents. Although some cases of IOI may resolve without impacting visual function, others, especially those linked to retinal vasculitis, including occlusive retinal vasculitis, can lead to severe and irreversible visual loss.^10,11^ Therefore, it is crucial for international pharmacovigilance agencies to proactively prevent, detect, and comprehend adverse events and other undesirable outcomes related to medications. Recently, cases of IOI with occlusive vasculitis associated with faricimab have been reported in eyes that underwent anti-VEGF therapy.^12,13^ Based on the data available as of the end of August 2023, the estimated reported rate of retinal vasculitis with occlusion is 0.06 cases per 10 000 injections of faricimab.^14,15^

In Switzerland, faricimab has been approved and reimbursed since May 25, 2022, with approximately 63 000 injections administered in the country to date. Notably, to our best knowledge, there have not been any reported cases of IOI-associated occlusive retinal vasculitis in Switzerland. The primary objective of this case series was to present and analyze instances of IOI associated with faricimab therapy that were referred to the ophthalmology department of the University Hospital of Zurich. This clinic acts as a tertiary referral center, covers an approximate population of 1.7 million individuals, and is one of the largest medical retina clinics in the country.

## Methods

### Study Design and Setting

This study was a single-center retrospective case series conducted in an academic-based tertiary referral center in Switzerland. General written, informed consent was obtained for all patients, and the study was approved by the Ethics Committee of the Canton of Zurich, Switzerland (project No. PB_2016_00264). The study was conducted in accordance with the tenets of the Declaration of Helsinki and followed the Strengthening the Reporting of Observational Studies in Epidemiology (STROBE) reporting guidelines.

### Study Cohort

Electronic medical records and imaging of patients who were referred for IOI to the retina service at the ophthalmology department of the University Hospital of Zurich between June 1, 2022, and March 5, 2024, were reviewed. Of those, only eyes that developed a sterile IOI potentially associated with faricimab IVIs were included in the case series and further analyzed. Participant race and ethnicity information was not included in this analysis, as the majority of the patients identified as White race.

### Data Collection

Clinical characteristics of the study cohort who developed an IOI were recorded including sex, age, laterality, medical history (including autoimmune disease), ocular history, lens status, intraocular pressure (IOP), symptoms described by the patient, anatomic location of the IOI (anterior, intermediate, and/or posterior), including keratic precipitates, and presence of any kind of vasculitis and vascular occlusion. Corrected visual acuity (VA) with autorefraction or current glasses was tested using the Early Treatment of Diabetic Retinopathy Study (ETDRS) charts and reported as an ETDRS letter score along with approximate Snellen equivalent.

Moreover, number and type of prior anti-VEGF IVI, number and dates of faricimab injections, lot number of the presumably causative faricimab injection before the adverse event, the last injection interval, and the multimodal imaging features were considered in the data analysis. Images including color fundus photographs, fluorescein angiograms (FAs), indocyanine green angiograms, and optical coherence tomography (OCT) were evaluated. For patients referred from a physician based outside the university hospital, clinical images were also requested and reviewed.

The process of differential diagnosis exclusion was conducted through rigorous testing for potential confounding clinical characteristics, thereby effectively eliminating alternative diagnostic possibilities.

### Statistical Analysis

Descriptive statistics of clinical characteristics for patients included in the study were reported as median and interquartile range (IQR) for continuous variable and number for categorical variables. Data were analyzed using R software, version 4.1.2 (R Project for Statistical Computing).

## Results

A total of 12 eyes from 7 patients (mean [SD] age, 73.3 [16.7] years; 4 female [57.1%]; 3 male [42.9%]) over 22 months (eFigure in Supplement 1) were included in this retrospective study as they presented with IOI associated with faricimab injections. Eyes were treated for either neovascular AMD or DME. Eleven eyes had received previous treatment with other anti-VEGF agents. In the DME group, there was 1 treatment-naive patient. No cases of infectious endophthalmitis after faricimab injection were observed.

Seven eyes (58.3%) with neovascular AMD developed IOI after faricimab injections. Of these, 2 eyes developed vasculitis, 4 eyes developed anterior uveitis and vitritis, and 1 eye developed anterior uveitis only. Of the 2 eyes that developed vasculitis, they had an occlusive form of vasculitis of the arteries and veins, leading to subsequent macular capillary nonperfusion and clinically relevant irreversible vision deterioration from 20/80 to 20/2000. Conversely, 5 eyes treated with faricimab for DME developed anterior uveitis and vitritis. No vasculitis was detected in patients with DME.

The overall median (IQR) number of faricimab injections before the IOI event was 3.5 (2.0-4.3), and the median (IQR) interval between the last faricimab injection and the inflammation was 28 (24-38) days. Three eyes were found to have increased IOP (≥30 mm Hg) at the time of the IOI diagnosis. None of the cases showed clinical signs of infectious endophthalmitis.^15^

### Moderate IOI

A total of 10 eyes (83.3%) from 6 patients developed moderate IOI after administration of faricimab anti-VEGF injections. Among these, 5 eyes were undergoing treatment for neovascular AMD, whereas the remaining eyes were treated for DME. The median (IQR) time for presentation of IOI was 28 (27-36) days from the most recent faricimab injection. The median (IQR) number of faricimab injections before the event was 4.0 (2.3-4.8). Symptoms of IOI onset included floaters, ocular discomfort, pain, and decreased visual acuity, and 6 eyes were completely asymptomatic and found to have IOI on routine follow-up examination. The median VA letter score at IOI presentation was 60, approximately 20/63 (IQR, 65 [20/50] to 56 [20/80]). Anterior chamber inflammation was observed in all 10 cases, characterized by the presence of keratic precipitates (KPs). Five eyes revealed an anterior chamber cell score of 0.5+ in the 1 × 1-mm slitlamp field according to the Standardization of Uveitis Nomenclature criteria.^16^ The remaining 5 eyes showed a score of greater than or equal to 1.5+. Additionally, vitreous cells were observed in 9 of the 10 eyes with moderate IOI. Treatment primarily involved topical corticosteroids in all cases and was discontinued on resolution of the IOI. None of the eyes with moderate IOI experienced a relevant decline in visual acuity (>5 letters) after local corticosteroid treatment. The median (IQR) IOP at the onset of the event was found to be 19 (16-28) mm Hg, and increased intraocular pressure of 30 mm Hg or higher was found in 3 eyes. Detailed clinical characteristics have been summarized in the Table.

**Table.** Baseline Patient Characteristics of the Study Cohort

Variable	Case No./sex/age, y											
	Case 1/female/83		Case 2/female/84		Case 3/female/68		Case 4/male/44		Case 5/male/62	Case 6/male/78	Case 7/female/94	
Eye	OD	OS	OD	OS	OD	OS	OD	OS	OS	OS	OD	OS
Diagnosis	nAMD	nAMD	nAMD	nAMD	DME	DME	DME	DME	DME	nAMD	nAMD	nAMD
Lens status	PFK	PFK	PFK	PFK	Phakic	Phakic	Phakic	Phakic	Phakic	Phakic	PFK	PFK
Symptoms at onset of issues	Floaters	Reduced VA	Ocular discomfort	Ocular discomfort	NA	NA	NA	NA	Pain, reduced VA	NA	Pain, reduced VA	NA
VA (ETDRS score) at onset of issues	58	21	85	85	74	65	61	84	56	44	2	60
Approximate Snellen equivalent	20/80	20/320	20/20	20/20	20/32	20/50	20/63	20/20	20/80	20/125	20/800	20/63
IOP	17	14	14,7	16	21	21	37	44	17	12	30	16
Anterior chamber inflammation (at first evaluation)^a^	KP, 2+ cells	KP, 2+ cells	KP, 1+ cells	KP, 1+ cells	KP, 0.5+ cells	KP, 0.5+ cells	KP, 1.5+ cells	KP, 1.5+ cells	KP, 0.5+ cells	KP, 0.5+ cells	KP, 2+ cells	KP, 0.5+ cells
Vitreous cells	Yes	Yes	Yes	Yes	Yes	Yes	Yes	Yes	Yes	No	Yes	Yes
Vasculitis	No	Occlusive vasculitis of both arteries and veins	Arterial nonocclusive vasculitis	No	No	No	No	No	No	No	No	No
Faricimab, No.	1	2	3	3	2	2	4	4	5	4	6	5
Type of injections before faricimab	Aflib	Aflib	Aflib (3×)	Aflib (7×)	Aflib (7×)	Aflib (7×)	Aflib (16×)	Aflib (16×)		Aflib (72×)	Aflib (11×)	Aflib (10×)
				Rani (2×)								
Interval from last faricimab intravitreal injection to diagnosis, d	59^a^	3^a^	38^a^	38^a^	27	27	28	28	13	28	5^a^	89^a^
Last faricimab lot No.	B1512	B1512	B1514	B1514	B1500B08	B1500B08	B1501B03	B1501B03	NA	B1502B17	B1524B19	B1524B19
Treatment	Systemic CS		Systemic CS		Local CS		Local CS		Local CS	Local CS	Local CS, later systemic	

Abbreviations: Aflib, aflibercept; CS, corticosteroid; DME, diabetic macular edema; ETDRS, Early Treatment Diabetic Retinopathy Study; IOP, intraocular pressure; KP, keratic precipitate; NA, not available; nAMD, neovascular age-related macular degeneration; PFK, pseudophakia; Rani, ranibizumab; VA, visual acuity.

^a^ First diagnosis at the private ophthalmologist.

### Severe IOI

#### Case 1

An 83-year-old female with bilateral pseudophakia and neovascular AMD was referred to the ophthalmic emergency service with a suspected diagnosis of bilateral anterior uveitis and concomitant central retinal vein occlusion. The patient had received an IVI of faricimab in her left eye 8 days before. The right eye was also under faricimab treatment and had the last IVI 59 days before referral.

**Figure 1.**
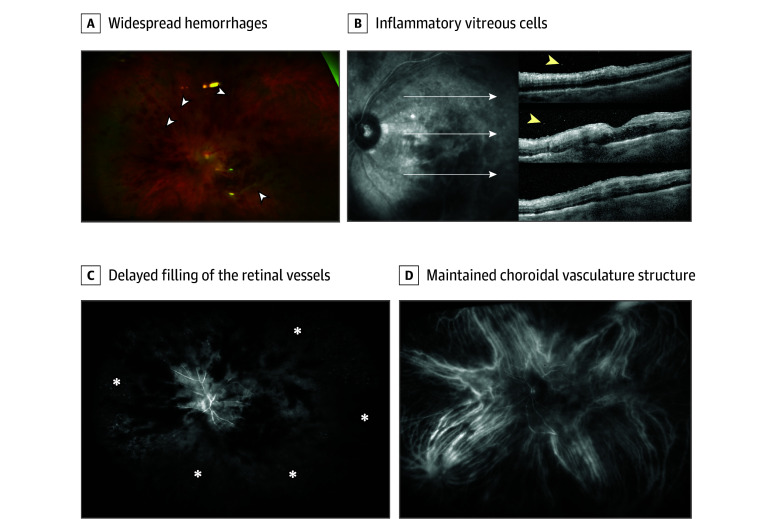
Baseline Multimodal Imaging of Case 1 A, Optos widefield pseudocolor image of the left eye of the patient in case 1 depicts widespread hemorrhages involving both the macular area and the periphery, with partial vitreous opacity including sheathing of the retinal arteries and veins (white arrowheads). B, Macular optical coherence tomography scans display the presence of inflammatory vitreous cells (yellow arrowheads) within the vitreous space and partial swelling of the outer nuclear layer. The inner retinal layers appear thin and disrupted. C, The early phase of fluorescein angiography (FA) clearly reveals diffuse intraretinal bleeding blocking the FA signal. Note the marked delayed filling of the retinal vessels leading to extensive diffuse vascular non-perfusion (white asterisks) in the periphery. D, Indocyanine green angiography portrays a maintained choroidal vasculature structure without clear pathological alterations.

Initial clinical examination revealed a decreased VA (−12 letters OD; − 36 letters OS) comparable with the VA detected on the last injection, without pain symptoms. Anterior segment examination showed diffuse endothelial KPs and anterior chamber cells bilaterally (2+). Fundus examination and fluorescein angiography confirmed the presence of an occlusive ocular vasculitis involving both arteries and veins only in the left eye characterized by the presence of diffuse hemorrhages, vascular leakage, optic nerve head hyperfluorescence, and extensive retinal capillary nonperfusion (Figure 1). The patient was admitted with suspicion of a possible bilateral faricimab-associated uveitis with ocular vasculitis component in the left eye. In addition, a comprehensive laboratory and radiological workup was performed to rule out any other secondary genesis of the uveitis.

The immunological, rheumatological, inflammatory, and infectious laboratory test results were unremarkable, except for positive IgG antibody detection for herpes simplex virus 1 (HSV1), HSV2, and varicella-zoster virus (VZV; IgM negative in each case), and the sonographic cardiovascular and cerebrovascular evaluation gave no indication of a relevant secondary genesis. Therefore, it was concluded that there was a potential sterile inflammatory component associated with faricimab. Consequently, treatment with systemic corticosteroids (1 mg/kg per day) was initiated.

**Figure 2.**
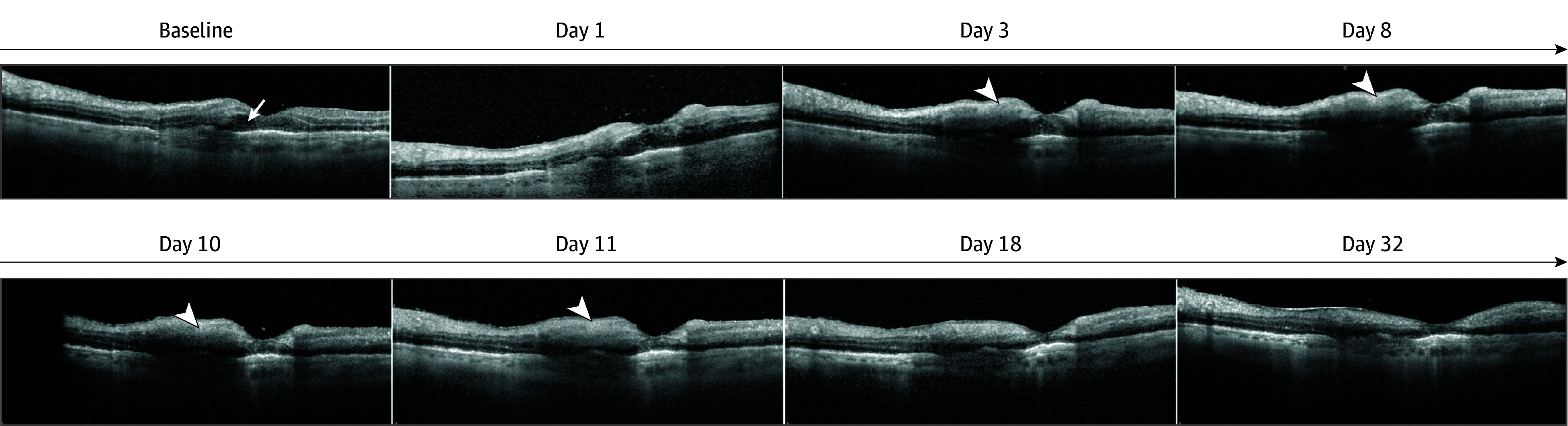
Case 1 Depicting a Foveal Optical Coherence Tomography Series The series of B-scans passing through the fovea show macular changes over time, starting with initial swelling including intraretinal fluid (white arrow), progressing through hyperreflectivity of the inner retinal layers (white arrowheads), and culminating in complete macular capillary nonperfusion after 32 days, resulting in the thinning of all retinal layers.

The left eye gradually progressed to macular capillary nonperfusion with a remarkable thinning of the inner retinal layers and an irreversible decline of the visual acuity (20/2000). The foveal OCT images are presented in Figure 2. Panretinal photocoagulation was also planned to treat peripheral capillary nonperfusion.

#### Case 2

**Figure 3.**
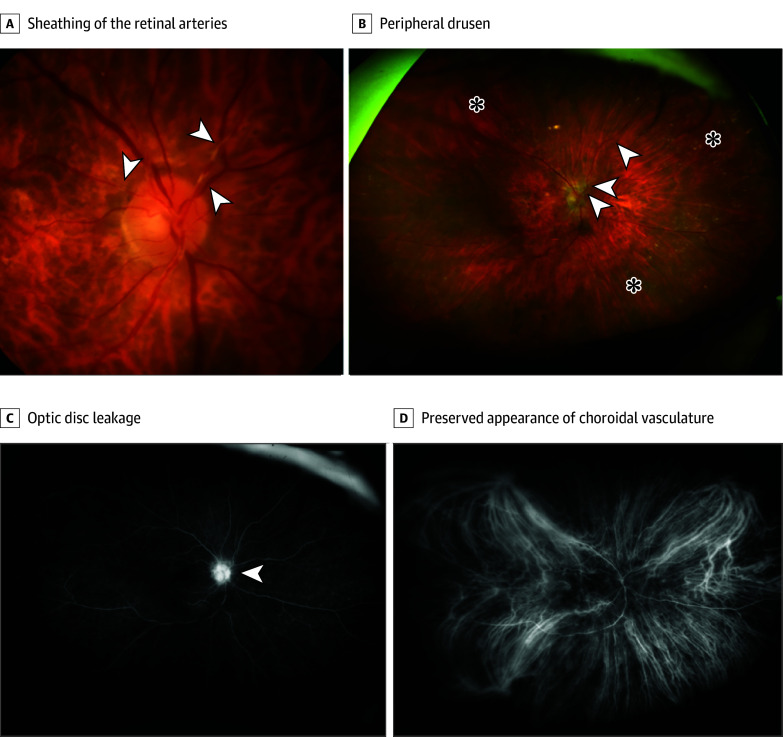
Right Eye Presentation of Baseline Case 2 Multimodal Imaging A, Flash-based color fundus photography clearly highlights the presence of sheathing of the retinal arteries (white arrowheads). B, This is confirmed by the widefield Optos image (arrowheads), which shows a large amount of peripheral drusen (asterisks). C, Fluorescein angiography clearly shows optic disc leakage (arrowhead) and macular staining of drusen and fibrovascular pigment epithelial detachment related to age-related macular degeneration but no areas of nonperfusion. D, Indocyanine green angiography reveals a preserved appearance of the choroidal vasculature and no signs of choroidal ischemia.

**Figure 4.**
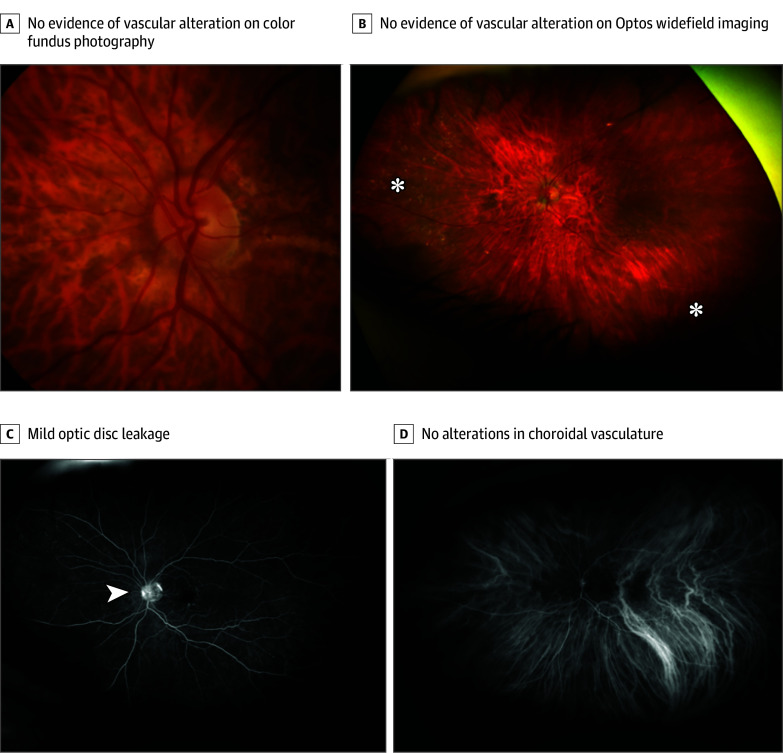
Left Eye Presentation of Baseline Case 2 Multimodal Imaging A and B, Color fundus photography and Optos widefield imaging, respectively, of the left eye demonstrate no evidence of vascular alteration associated with vasculitis. B, Peripheral drusen (asterisks) appear symmetric to those in the right eye. C, Fluorescein angiography reveals mild optic disc leakage (arrowhead), drusen staining in the macula associated with age-related macular degeneration, and drusen staining in the periphery. D, Indocyanine green angiography depicts no particular alterations in choroidal vasculature, similar to the findings in the fellow eye.

An 84-year-old female patient was referred by her private ophthalmologist due to a progressive decrease in visual acuity since November 2023, accompanied by a new manifestation of bilateral endothelial KPs and anterior cells (2+) after having had the third faricimab IVI in both eyes 38 days before referral to our hospital. The patient’s ophthalmological history included neovascular AMD, bilateral pseudophakia, and she was status post bilateral YAG iridotomy due to a plateau iris configuration. The patient’s general systemic medical history was otherwise unremarkable, with no evidence of systemic comorbidities or etiopathogenesis underlying the ophthalmological symptoms. The external ophthalmologist who administered the injection had already initiated bilateral topical corticosteroid treatment, which was then discontinued on initiation of systemic corticosteroid therapy in our hospital. The clinical examination confirmed the findings, with the addition of a mild bilateral vitreous inflammation (right eye greater than left), perivascular sheathing of the peripapillary arterial vessels in the right eye, and visual acuity with a letter score of 85 (20/20 OU) on the ETDRS chart. IOP was found below normal tension values (<18 mm Hg) in both eyes. To further investigate and expand the ophthalmological diagnosis, FA and OCT evaluations were performed, initially showing only mild optic nerve head hyperfluorescence in the right eye without evidence of occlusive or exudative changes (Figure 3 and Figure 4). Suspecting faricimab-associated ocular inflammation/vitritis, the patient’s physician had her undergo a comprehensive radiological and laboratory evaluation of potential etiopathogenic factors. Immunological, rheumatological, inflammatory, and infectious laboratory tests were unremarkable, except for positive IgG antibody detection for HSV1, HSV2, and VZV (IgM negative in each case), and the sonographic cardiovascular and cerebrovascular evaluation gave no indication of a relevant secondary genesis. Therefore, it was concluded that there was a potential sterile inflammatory component associated with faricimab. Consequently, treatment with systemic corticosteroids (1 mg/kg per day) was initiated 40 days after the last faricimab injection. Further in the course of the examination, a stable constellation of findings was observed, with fluctuating but overall stable VA and IOP parameters, allowing for an appropriate tapering schedule of systemic corticosteroid therapy to be initiated.

## Discussion

We present a case series of noninfectious (sterile) IOI associated with intravitreal injections of faricimab in patients affected by neovascular AMD and DME. Among the eyes evaluated, 2 exhibited retinal vasculitis, including both eyes of 1 case with an occlusive vasculitis resulting in irreversible vision-threatening sequelae and consequent macular capillary nonperfusion in that case.

In the TENAYA (A Phase III, Multicenter, Randomized, Double-Masked, Active Comparator-Controlled Study to Evaluate the Efficacy and Safety of Faricimab in Patients With Neovascular Age-Related Macular Degeneration) and LUCERNE (A Phase III, Multicenter, Randomized, Double-Masked, Active Comparator-Controlled Study to Evaluate the Efficacy and Safety of Faricimab in Patients With Neovascular Age-Related Macular Degeneration) trials, the 2 phase 3 RCTs of neovascular AMD, the IOI rate was 2.0% among patients receiving faricimab, with no reported cases of occlusive vasculitis in either study.^2^ Similarly, the phase 3 RCTs, YOSEMITE (A Phase III, Multicenter, Randomized, Double-Masked, Active Comparator-Controlled Study to Evaluate the Efficacy and Safety of Faricimab [RO6867461] in Patients With Diabetic Macular Edema) and RHINE (A Phase III, Multicenter, Randomized, Double-Masked, Active Comparator-Controlled Study to Evaluate the Efficacy and Safety of Faricimab [RO6867461] in Patients With Diabetic Macular Edema) for DME, yielded comparable results, with IOI rates ranging from 0.6% to 2.2% in the faricimab treatment arms.^3^ Our case series was not designed to collect incident data, and thus we cannot directly compare our findings with those of RCTs. However, based on the drug warning released by Genentech in November 2023,^11^ there are spontaneous postmarketing reports of retinal vasculitis with or without occlusion in patients treated with faricimab.

Like the RCTs, clinical practice setting studies demonstrated an overall safety profile of faricimab treatment in patients with neovascular AMD and DME, with an inflammation rate comparable with similar treatments and no cases of retinal vasculitis or artery occlusion reported.^17,18^

Recently, Thangamathesvaran et al^15^ reported culture-negative vitreous taps of 3 cases of severe IOI after intravitreal injections of faricimab, originating from 3 distinct clinic locations. Interestingly, these cases manifested acute and severe anterior and posterior inflammation, hindering confident evaluation of the retinal vasculature. This observation raises the possibility that the vitreous inflammation may have obscured signs of active vasculitis, thus contributing to the absence of detectable vasculitis after inflammation resolved.

Momenaei et al^19^ retrospectively reported rates of ocular adverse events after intravitreal faricimab injections at a single site in the US. They observed IOI in 1% of treated eyes with no prior history of uveitis. Dedicated examinations, such as FA, revealed no evidence of occlusive retinal vasculitis or retinal artery occlusion. The overall occurrence of IOI was comparable with that observed in previous phase 3 trials.

Uveitis secondary to faricimab administration was documented in 3 eyes of 2 patients by Palmieri et al.^14^ They presented a case series detailing noninfectious IOI in 2 female patients with diabetes exhibiting granulomatous uveitis, accompanied by elevated IOP, without evidence of vasculitis and occlusion-related events.^14^

More recently, Li et al^20^ documented the first case of bilateral occlusive vasculitis associated with bilateral intravitreal faricimab injections administrated 18 days earlier. The authors presented a case of a 96-year-old-woman with neovascular AMD who began to experience pain and vision decline 3 days after bilateral therapy.

The patients in the current study developed IOI after a median of 3.5 faricimab injections, ranging from 1 to a maximum of 6 injections before the event. These data are consistent with available case series and clinical practice setting studies, where IOI typically manifests during or shortly after the loading dose phase.^14,17,19^

The interval between the last faricimab injection and the IOI diagnosis varies from 3 to 90 days, with a median of 28 days before the inflammation occurs. This wide range can be attributed to the occurrence of bilateral asymmetric inflammation in some cases, where 1 eye exhibited more severe symptoms and was therefore evaluated more promptly. Consequently, the interval between the last injection and the occurrence of IOI might be overestimated. Data from clinical practice setting studies are warranted to better elucidate this important aspect.

IOI complicated by retinal vasculitis has been extensively reported in the past after anti-VEGF injections and more recently in cases after intravitreal pegcetacoplan administration for geographic atrophy.^21,22,23,24^

Brolucizumab treatment has been found to be related to a higher incident of retinal vasculitis and/or retinal vascular occlusion as postmarketing surveillance data demonstrated a rate of 15.5 events per 10 000 injections worldwide in neovascular AMD.^25^

Brolucizumab was initially approved for the treatment of neovascular AMD and DME without any particular concern, as clinical trial data yielded promising results regarding safety.^26,27,28^ However, only after widespread use in several countries and subsequent publication of clinical practice setting data analysis, the drug’s safety profile was reconsidered and analyzed in light of severe IOI cases reported in the scientific literature.^22,29,30^ It is noteworthy that faricimab has been available on the international market for over a year with more than 2.5 million vials distributed worldwide, with only a few reported cases of severe vasculitis and/or occlusive retinal vascular events. Similarly, pegcetacoplan treatment for geographic atrophy has exhibited a favorable safety profile, with only a handful of severe IOI cases published to date.^24,31,32^ However, it is pertinent to mention that pegcetacoplan remains accessible in only a few countries; for instance, it was declined by the European Medicines Agency and other drug regulatory agencies. Therefore, clinical practice setting long-term data are limited.

### Limitations

This case series is influenced by several limitations that warrant acknowledgment. First, a case series cannot determine a cause-and-effect relationship. Potential faricimab-associated IOI will inevitably require further investigation. Second, it is possible that some moderate cases of IOI may have gone undetected, as the patients could have been diagnosed and treated at other health care facilities without referral to a tertiary care center. Third, faricimab treatments may have been administered by different physicians within the Canton of Zurich, making it challenging to accurately estimate and compare the incidence rates of IOI events among patients undergoing treatment and the number of injections administered. Lastly, the retrospective design of this analysis introduces intrinsic biases, which may limit our ability to establish a temporal relationship between exposure to treatment and the IOI outcome.

## Conclusions

In conclusion, this retrospective study presented a series of intraocular inflammation cases after faricimab intraocular injections. Within this series, 2 eyes developed vasculitis, and 1 eye also experienced occlusion-related retinopathy, resulting in irreversible vision loss. In all cases, a sterile inflammation was suspected as there were no indicators for an infectious disease during the etiologic workup. Although these findings do not establish causality and can only generate hypotheses for future investigations, they underscore the importance of maintaining a high level of vigilance and attention when introducing new medications into routine clinical practice, especially when their safety profiles are not yet well established. Imaging is crucial for identifying and assessing the extent of inflammation, and prompt corticosteroid treatment tailored to the severity of the inflammatory response is recommended. Further data, derived from multicenter studies, are essential to provide additional insights into the safety and efficacy of faricimab in a larger patient population and to create clinical practice setting evidence. This is particularly important considering the restrictive inclusion criteria often used in RCTs, which may limit the generalizability of their safety findings.

## Notes

Supplement 1. **eFigure.** Distribution of Intraocular Inflammation (IOI) Events Over 22 Months

Supplement 2.
Data Sharing Statement.
