# Supplementary material for: Sterile Intraocular Inflammation Associated With Faricimab
Source: JAMA Ophthalmol. 2024 Oct 10;142(11):1028–36. doi: 10.1001/jamaophthalmol.2024.3828 (PMC11581583; doi:10.1001/jamaophthalmol.2024.3828)
Supplement: Supplement 1. — eFigure. Distribution of Intraocular Inflammation (IOI) Events Over 22 Months [file jamaophthalmol-e243828-s001.pdf]

Supplementary Online Content

Cozzi M, Ziegler A, Fasler K, Muth DR, Blaser F, Zweifel S. Sterile intraocular inflammation associated with faricimab. *JAMA Ophthalmol*. Published online October 10, 2024. doi:10.1001/jamaophthalmol.2024.3828

eFigure. Distribution of Intraocular Inflammation (IOI) Events Over 22 Months

This supplementary material has been provided by the authors to give readers additional information about their work.

© 2024 Cozzi M et al. *JAMA Ophthalmology.*

eFigure. Distribution of Intraocular Inflammation (IOI) Events Over 22 Months

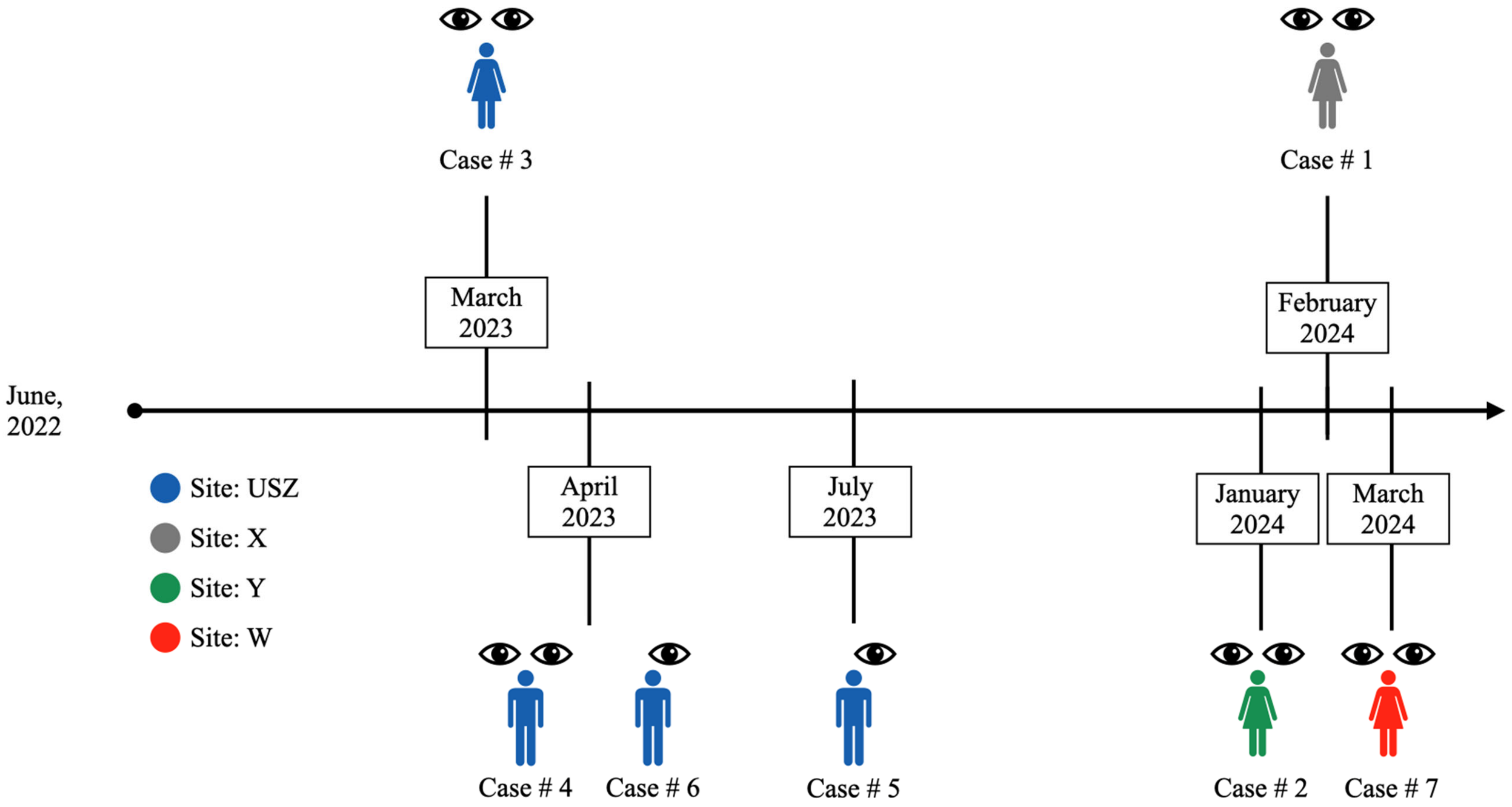

This figure represents the occurrence of IOI events over a 22-months period. Notably, different colors indicate the various clinics where the patients were treated.

© 2024 Cozzi M et al. *JAMA Ophthalmology.*
