# Supplementary material for: Sterile Intraocular Inflammation Associated With Faricimab
Source: JAMA Ophthalmol. 2024 Oct 10;142(11):1028–36. doi: 10.1001/jamaophthalmol.2024.3828 (PMC11581583; doi:10.1001/jamaophthalmol.2024.3828)
Supplement: Supplement 2. — Data Sharing Statement. [file jamaophthalmol-e243828-s002.pdf]

Data Sharing Statement

Cozzi. Sterile Intraocular Inflammation Associated With Faricimab. *JAMA Ophthalmol*.
Published October 10, 2024. doi:10.1001/jamaophthalmol.2024.3828

Data
Data available: No
